# Panacea for Trouble

**Published:** 1885-01

**Authors:** 


### Panacea for Trouble.

Life is filled with trouble, as a writer in *Our Homes* has said, and we must shoulder our share with the best grace we can. We may only seek to make them as light as we can, since to avoid them is impossible.

There is one sovereign panacea for this. It is work. Brooding over trouble is like surrounding one's self with a fog. It magnifies all objects seen through it. Occupation of the mind prevents this; hard work, manual work even, gives the mind other matters of concern, tires the body so that sleep will come.

Very few suicides occur when men are actively employed. When out of work they think of their other troubles, and the despondency arising from this added one throws the mind from its balance, and the fatal deed is done. Many a man would have committed suicide if he had had the time. Work of any kind, especially work for others, is the great panacea for a troubled mind.

